# Bone morphogenetic protein 9 (BMP9) and BMP10 enhance tumor necrosis factor-α-induced monocyte recruitment to the vascular endothelium mainly via activin receptor-like kinase 2

**DOI:** 10.1074/jbc.M117.778506

**Published:** 2017-06-23

**Authors:** Claudia-Gabriela Mitrofan, Sarah L. Appleby, Gerard B. Nash, Ziad Mallat, Edwin R. Chilvers, Paul D. Upton, Nicholas W. Morrell

**Affiliations:** From the ‡ Department of Medicine, University of Cambridge School of Clinical Medicine, Cambridge CB2 0QQ and; the § Institute of Cardiovascular Sciences, College of Medical and Dental Sciences, University of Birmingham, Edgbaston, Birmingham B15 2TT, United Kingdom

**Keywords:** atherosclerosis, bone morphogenetic protein (BMP), endothelial cell, monocyte, SMAD transcription factor

## Abstract

Bone morphogenetic proteins 9 and 10 (BMP9/BMP10) are circulating cytokines with important roles in endothelial homeostasis. The aim of this study was to investigate the roles of BMP9 and BMP10 in mediating monocyte–endothelial interactions using an *in vitro* flow adhesion assay. Herein, we report that whereas BMP9/BMP10 alone had no effect on monocyte recruitment, at higher concentrations both cytokines synergized with tumor necrosis factor-α (TNFα) to increase recruitment to the vascular endothelium. The BMP9/BMP10-mediated increase in monocyte recruitment in the presence of TNFα was associated with up-regulated expression levels of E-selectin, vascular cell adhesion molecule (VCAM-1), and intercellular adhesion molecule 1 (ICAM-1) on endothelial cells. Using siRNAs to type I and II BMP receptors and the signaling intermediaries (Smads), we demonstrated a key role for ALK2 in the BMP9/BMP10-induced surface expression of E-selectin, and both ALK1 and ALK2 in the up-regulation of VCAM-1 and ICAM-1. The type II receptors, BMPR-II and ACTR-IIA were both required for this response, as was Smad1/5. The up-regulation of cell surface adhesion molecules by BMP9/10 in the presence of TNFα was inhibited by LDN193189, which inhibits ALK2 but not ALK1. Furthermore, LDN193189 inhibited monocyte recruitment induced by TNFα and BMP9/10. BMP9/10 increased basal IκBα protein expression, but did not alter p65/RelA levels. Our findings suggest that higher concentrations of BMP9/BMP10 synergize with TNFα to induce the up-regulation of endothelial selectins and adhesion molecules, ultimately resulting in increased monocyte recruitment to the vascular endothelium. This process is mediated mainly via the ALK2 type I receptor, BMPR-II/ACTR-IIA type II receptors, and downstream Smad1/5 signaling.

## Introduction

The vascular endothelium is a key regulator of vascular homeostasis with important roles in regulating blood pressure, coagulation, leukocyte trafficking, and angiogenesis (1–3). The normal vascular endothelium regulates the passage of circulating cells into the interstitial space through several mechanisms, including leukocyte recruitment and alterations in permeability. However, endothelial dysfunction initiates a series of events triggering aberrant endothelial activation that can lead to chronic pathological permeability and leukocyte adherence (4), which contribute to cardiovascular diseases, including atherosclerosis.

Chronic systemic inflammation is associated with many cardiovascular, rheumatological, and respiratory diseases (5–7), principally through the pathological activation of the vascular endothelium. Inflammatory cytokines including tumor necrosis factor α (TNFα) and interleukin-1β are elevated in atherosclerosis. This promotes the up-regulation of endothelial-expressed cell surface proteins that mediate leukocyte adhesion, including P- and E-selectin, which are involved in the initial leukocyte capture, and intercellular adhesion molecule 1 (ICAM-1),^4^ and vascular cell adhesion molecule 1 (VCAM-1), which regulate the firm adhesion and transmigration of leukocytes (8–10).

Bone morphogenetic proteins (BMPs) are ligands belonging to the TGFβ superfamily. Aberrant BMP2, BMP4, and BMP6 signaling have been associated with the inflammation, fibrosis, calcification, and osteogenesis that are associated with the pathophysiology of atherosclerosis (11–17). Because BMP9 and BMP10 are potent mediators of endothelial function it is likely that they also contribute to the pathobiology of vascular diseases such as atherosclerosis. However, the role played by BMP9 and BMP10 in monocyte transmigration across the endothelium, one of the initiating steps in atherosclerosis, has not been studied. BMP9 is a key regulator of vascular quiescence (18, 19), and has been shown to protect the endothelium through the inhibition of vascular permeability (20), endothelial proliferation (18), angiogenesis (21), and lymphangiogenesis (22, 23). Although BMP9 has been more extensively characterized than BMP10, in cell culture experiments BMP10 regulates a similar set of genes as BMP9 (24) and BMP10 can substitute for BMP9 in a mouse model of postnatal retinal vascular remodeling (21). Moreover, similar to BMP9, BMP10 has been described as a mediator of flow-dependent arterial quiescence (25). These studies suggest an overlapping role and function for BMP9 and BMP10 in the vasculature.

BMP serine-threonine kinase receptors form heterodimeric complexes consisting of type I and type II receptors (26). BMP9 and BMP10 signal through type I and type II receptors expressed on endothelial cells, including the type I receptors, activin-like kinase (ALK) 1 and ALK2, and the type II receptors, bone morphogenetic protein receptor 2 (BMPR-II encoded by *BMPR2*), activin receptor 2A (ACTR-IIA encoded by *ACVR2A*), and activin receptor 2B (ACTR-IIB encoded by *ACVR2B*) (24, 27, 28). Optimal BMP9 and BMP10 signaling requires the type III auxiliary receptor endoglin, also expressed on endothelial cells (27). Mutations in BMP9 and its' receptors underlie vascular diseases, namely hereditary hemorrhagic telangiectasia (ALK1, endoglin, and BMP9) (29–31) and pulmonary arterial hypertension (ALK1, BMPR2) (32–34). Furthermore, endothelial deletion of *Bmpr2* in mice enhances the development of atherosclerosis, suggesting an atheroprotective protective role for BMPR-II (35).

Activated BMP receptors transduce their signal primarily through phosphorylation of Smad1, Smad5, and Smad8. Following activation, Smads form heteromeric complexes with the common partner Smad, Smad4 (26). These complexes translocate to the nucleus and regulate the expression of numerous genes through binding to promoter regions, usually in complex with other transcription factors. The best characterized targets of BMP/Smad signaling are the inhibitor of differentiation (*ID*) genes, which possess Smad-binding elements in their promoters (36).

BMP9 signaling has been implicated previously in neutrophil recruitment to the endothelium, both directly (37) and indirectly (38–40). BMP9 has previously been shown to up-regulate E-selectin and VCAM-1 on LPS-stimulated blood outgrowth endothelial cells (37) and endothelial cell surface-expressed endoglin enhances leukocyte recruitment through the activation of β1-integrins expressed on the surface of leukocytes (40). Furthermore, *BMPR2*-deficient endothelium shows impaired leukocyte recruitment (38, 39), thus further implicating BMP9 signaling in the process of leukocyte recruitment.

Monocyte recruitment to the vascular endothelium is a key mediator of the progression of atherosclerotic lesions (41, 42). Although there is a growing body of evidence associating BMP9 signaling with neutrophil recruitment, the role of BMP9 and BMP10 in monocyte recruitment to the vascular endothelium has yet to be reported. In the current study we show, using an *in vitro* flow adhesion assay that both BMP9 and BMP10, in a concentration-dependent manner, synergistically enhance monocyte recruitment to TNFα-stimulated human aortic endothelial cells (HAECs). This occurs through the up-regulation of E-selectin, VCAM-1, and ICAM-1 on HAECs, and mainly via the type I receptor ALK2, the type II receptors BMPR-II/ACTR-IIA, and the downstream mediators Smad1/5.

## Results

### BMP9 and BMP10 increase monocyte recruitment to TNFα-treated HAECs in a concentration-dependent manner

**Figure 1.**
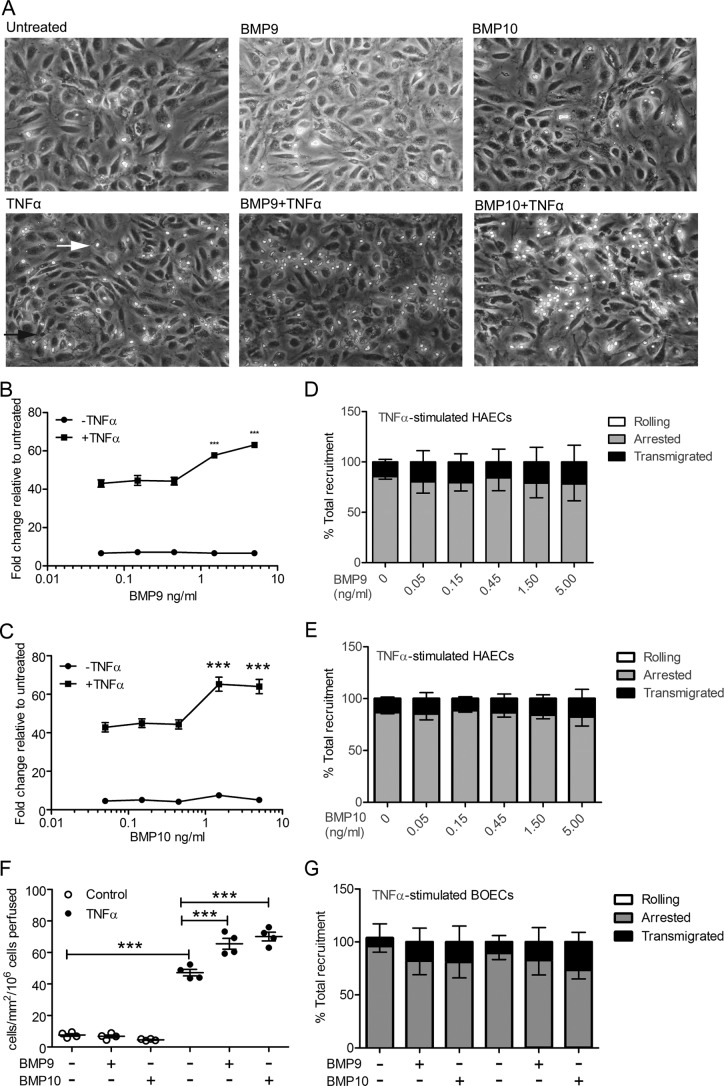
**BMP9 and BMP10 increase monocyte recruitment to TNFα-stimulated HAECs in a concentration-dependent manner.** HAECs were treated with BMP9 and BMP10 16 h prior to TNFα treatment (0.05 ng/ml, 4 h). Monocytes were perfused over TNFα-stimulated HAECs in a flow adhesion assay in the presence of media alone, BMP9 or BMP10. *A,* representative images of HAEC monolayers that were untreated, or treated with BMP9 (5 ng/ml), BMP10 (5 ng/ml), TNFα, BMP9 + TNFα, or BMP10 + TNFα. Adherent monocytes are the bright phase cells (*white arrow*) and transmigrated monocytes shown as the smaller dark phase cells (*black arrow*). Experiments were performed in triplicate and the data are representative of *n* = 3 biological repeats. *B* and *C,* concentration-response analysis of the recruitment of monocytes to HAEC monolayers, in the presence or absence of TNFα, with increasing concentrations of BMP9 (*B*) (0–5 ng/ml) and BMP10 (*C*) (0–5 ng/ml). *D* and *E*, monocyte behavior (rolling, *clear bar*; adherence, *gray bar*; and transmigration, *black bar*) was expressed as a percentage of total recruitment to TNFα-stimulated HAECs in the presence of BMP9 (*D*) and BMP10 (*E*). *F,* analysis of the recruitment of monocytes to BOEC monolayers, treated with 5 ng/ml of BMP9 or BMP10, in the presence or absence of TNFα. *G,* monocyte behavior (rolling, *clear bar*; adherence, *gray bar*; and transmigration, *black bar*) was expressed as a percentage of total recruitment to TNFα-stimulated BOECs in the presence of BMP9 or BMP10. *Error bars* represent ± S.E. *, *p* ≤ 0.05; **, *p* ≤ 0.01; ***, *p* ≤ 0.001.

First, we investigated the role of BMP9 and BMP10 on monocyte recruitment to the vascular endothelium using an *in vitro* flow adhesion assay, which enables the quantification of real-time interactions between endothelial cells and leukocytes under conditions of physiological flow. As BMP9 has been reported to circulate at concentrations between 2 and 12 ng/ml in humans (18, 43), we exposed the endothelium to BMP9 or BMP10 at concentrations ranging from 0 to 5 ng/ml prior to the addition of TNFα, then assessed monocyte recruitment. Negligible monocyte recruitment was observed in HAECs treated with BMP9 (Fig. 1, *A* and *B*) or BMP10 (Fig. 1, *A* and *C*) alone. Although TNFα treatment, as previously reported (2, 44, 45), induced some monocyte recruitment to HAEC monolayers (Fig. 1, *A–C*), a synergistic increase in total monocyte recruitment was observed when TNFα-stimulated HAECs were pre-treated with BMP9 or BMP10 at concentrations equal to or higher than 1.5 ng/ml (Fig. 1, *A–C*). Pre-treatment of the vascular endothelium with BMP9 or BMP10 did not affect the percentage of rolling, arrested, or transmigrated monocytes (Fig. 1, *D* and *E*). Only minimal monocyte rolling was observed in these experiments, suggesting that this process is rapidly followed by arrest and transmigration. Maximal monocyte recruitment was observed when TNFα-stimulated HAECs were pre-treated with BMP9 or BMP10 at a concentration of 5 ng/ml, consequently, this concentration was used in all subsequent experiments. To examine whether this response was restricted to aortic cells, we also assessed the influence of BMP9 and BMP10 on TNFα-dependent recruitment of monocytes to blood outgrowth endothelial cell (BOEC) monolayers (46). Similar to HAECs, BMP9 and BMP10 did not influence monocyte adhesion to BOECs, but enhanced the recruitment observed in response to TNFα (Fig. 1, *F* and *G*). Taken together, these data show that both BMP9 and BMP10 synergize with TNFα to enhance monocyte recruitment to the vascular endothelium in a concentration-dependent manner, at or above 1.5 ng/ml.

### BMP9 and BMP10 increase expression of adhesion molecules and BMP2 in TNFα-treated HAECs

**Figure 2.**
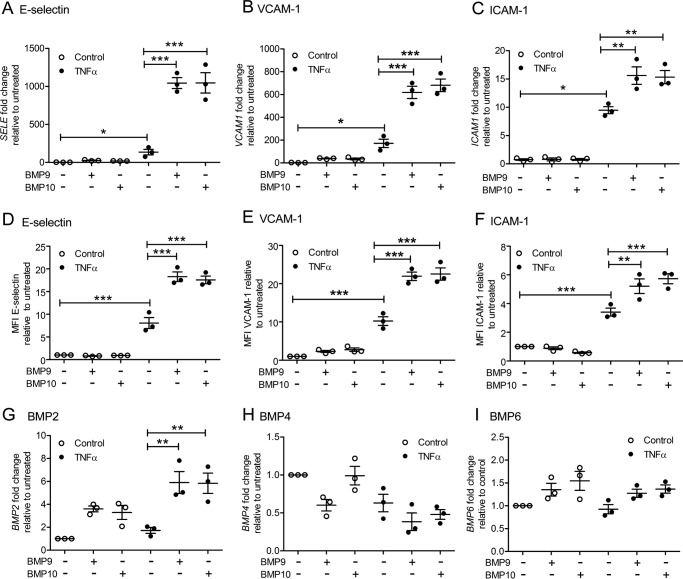
**BMP9 and BMP10 increase the expression of E-selectin, VCAM-1, ICAM-1, and BMP2 in TNFα-stimulated HAECs.** HAECS were treated with BMP9 or BMP10 (5 ng/ml, 16 h) prior to TNFα treatment (0.05 ng/ml, 4 h). Expression of *SELE* (E-selectin) (*A*), *VCAM1* (*B*), and *ICAM1* (*C*) mRNA assessed using qPCR. Surface expression of E-selectin (FITC-conjugated anti-human E-selectin) (*D*), VCAM-1 (PE-Cy5-conjugated anti-human VCAM-1) (*E*), and ICAM-1 (APC-conjugated anti-human ICAM-1) (*F*) was assessed using flow cytometry. Data are shown as median fluorescence intensity (*MFI*) expressed as fold-change relative to untreated HAECs. Expression of *BMP2* (*G*), *BMP4* (*H*), and *BMP6* (*I*) mRNA assessed using qPCR. Experiments were performed in triplicate and the data are representative of *n* = 3 biological repeats. *Error bars* represent mean ± S.E. *, *p* ≤ 0.05; **, *p* ≤ 0.01; ***, *p* ≤ 0.001.

Next, we used quantitative PCR (qPCR) and flow cytometry to identify whether pre-treatment with BMP9 or BMP10 increased expression of the endothelial selectins and adhesion molecules involved in monocyte recruitment in TNFα-stimulated HAECs. In accordance with previous studies (47–50), TNFα induced gene and surface protein expression of E-selectin, VCAM-1, and ICAM-1, which were synergistically increased in HAECs (Fig. 2, *A–F*, and supplemental Fig. S1) or BOECs (supplemental Fig. S2, *A–C*) pre-treated with either BMP9 or BMP10 (5 ng/ml). BMP9 and BMP10 alone had no effect on the expression of these adhesion molecules. P-selectin was not detected on HAECs with any of the treatments (data not shown).

Because BMP2, BMP4, and BMP6 have been previously implicated in inflammation, fibrosis, and osteogenesis (11–13), we next investigated whether treatment with BMP9 or BMP10 increased expression of these ligands in HAECs. BMP9 and BMP10 alone induced the expression of *BMP2* by 3–4-fold in HAECs, whereas TNFα exerted a weak induction (Fig. 2*G*). However, pre-treatment with either BMP9 or BMP10 prior to TNFα stimulation accentuated *BMP2* expression in HAECs (Fig. 2*G*). *BMP4* was slightly repressed by BMP9, BMP10, and TNFα, whereas *BMP6* expression did not change with any of the conditions tested (Fig. 2, *H* and *I*). Taken together, these data reveal that both BMP9 and BMP10 synergize with TNFα to up-regulate endothelial-expressed molecules involved in leukocyte recruitment, in addition to *BMP2*, a factor previously implicated in endothelial inflammation.

### BMP6 increases the surface expression of adhesion molecules on TNFα-treated HAECs

**Figure 3.**
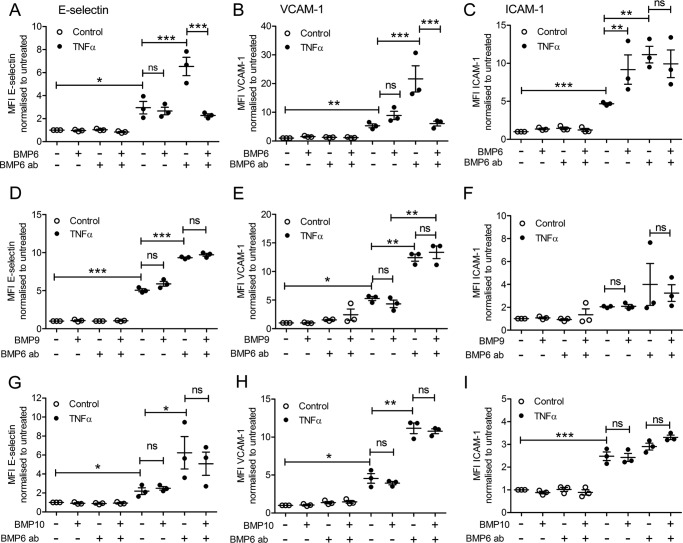
**BMP6 increases the expression of E-selectin, VCAM-1, and ICAM-1 in TNFα-stimulated HAECs.** HAECS were treated with a BMP6 neutralizing antibody (*BMP6 ab*) 60 min prior to the addition of BMP6 (25 ng/ml, 16 h) followed by TNFα (0.05 ng/ml; 4 h). Surface expression of E-selectin (FITC-conjugated anti-human E-selectin) (*A*), VCAM-1 (PE-Cy5-conjugated anti-human VCAM-1) (*B*), and ICAM-1 (APC-conjugated anti-human ICAM-1) (*C*) was assessed using flow cytometry. HAECs were treated with a BMP6 neutralizing antibody (*BMP6 ab*) 60 min prior to the addition of BMP9 (5 ng/ml, 16 h) followed by TNFα (0.05 ng/ml, 4 h). Surface expression of E-selectin (FITC-conjugated anti-human E-selectin) (*D*), VCAM-1 (PE-Cy5-conjugated anti-human VCAM-1) (*E*), and ICAM-1 (APC-conjugated anti-human ICAM-1) (*F*) was assessed using flow cytometry. HAECs were treated with a BMP6 neutralizing antibody (*BMP6 ab*) 60 min prior to the addition of BMP10 (5 ng/ml, 16 h) followed by TNFα (0.05 ng/ml, 4 h). Surface expression of E-selectin (FITC-conjugated anti-human E-selectin)(*G*), VCAM-1 (PE-Cy5-conjugated anti-human VCAM-1)(*H*), and ICAM-1 (APC-conjugated anti-human ICAM-1) (*I*) was assessed using flow cytometry. Forward scatter and side scatter gating was applied to the HAEC population. Data are shown as median fluorescence intensity (*MFI*) expressed as fold-change relative to untreated HAECs. Experiments were performed in triplicate and the data are representative of *n* = 3 biological repeats. *Error bars* represent mean ± S.E. *, *p* ≤ 0.05; **, *p* ≤ 0.01; ***, *p* ≤ 0.001; *ns*, not significant.

BMP6 has been previously described as a factor that induces endothelial inflammation (11). BMP6 transduces signaling predominantly via the type I receptor ALK2, and not ALK1 (11, 51). Therefore, we investigated the potential role of BMP6/ALK2 in inducing E-selectin, VCAM-1, and ICAM-1 surface expression in HAECs. BMP6 pre-treatment induced a marked up-regulation of the surface expression levels of E-selectin, VCAM-1, and ICAM-1 in TNFα-treated HAECs (Fig. 3, *A–C*). The up-regulation in E-selectin and VCAM-1 in response to BMP6 was completely abrogated by the use of a neutralizing anti-BMP6 antibody (Fig. 3, *A* and *B*). Treatment with the BMP6-targeted antibody did not cause any further reduction in ICAM-1 (Fig. 3*C*), indicating that ICAM-1 surface protein expression is regulated through a different mechanism to E-selectin and VCAM-1.

We next determined whether the BMP9/BMP10-induced up-regulation of adhesion molecules was mediated by BMP6. Treatment with the anti-BMP6 neutralizing antibody likewise had no effect on the surface expression levels of adhesion molecules induced by BMP9, BMP10, and TNFα treatments, indicating that this process was not mediated by BMP9 or BMP10 (Fig. 3, *D–H*). Collectively, these data imply a dominant role for ALK2-mediated effects of BMPs in the up-regulation of surface expression levels of endothelial adhesion molecules.

### The role of type I receptors in BMP9- and BMP10-induced expression of adhesion molecules

**Figure 4.**
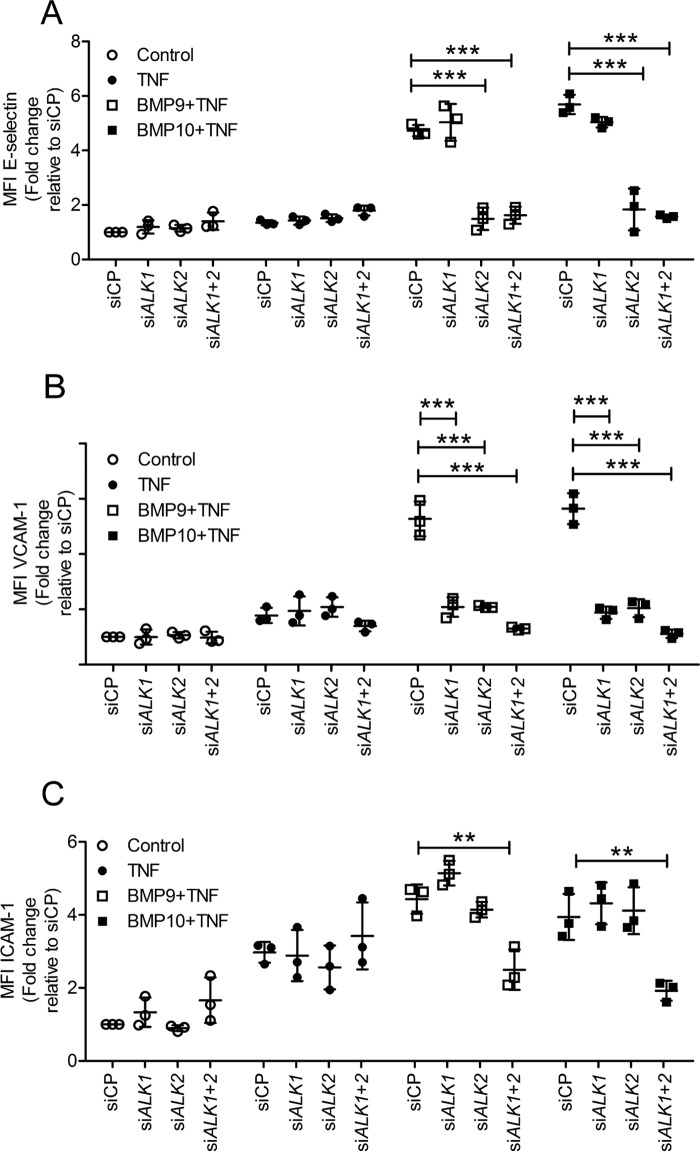
**Effect of ALK1 and ALK2 siRNA on BMP9- and BMP10-induced up-regulation of E-selectin, VCAM-1, and ICAM-1 in TNFα-stimulated HAECs.** HAECs were siRNA transfected, then treated with BMP9 or BMP10 (5 ng/ml; 16 h) prior to TNFα treatment (0.05 ng/ml for 4 h). Surface expression of E-selectin (FITC-conjugated anti-human E-selectin) (*A*), VCAM-1 (PE-Cy5-conjugated anti-human VCAM-1) (*B*), and ICAM-1 (APC-conjugated anti-human ICAM-1) (*C*) was assessed using flow cytometry. Forward scatter and side scatter gating was applied to the HAEC population. Data are shown as median fluorescence intensity (*MFI*) expressed as fold-change relative to HAECs transfected with siRNA control pool (*siCP*). Experiments were performed in triplicate and the data are representative of *n* = 3 biological repeats. *Error bars* represent mean ± S.E. ***, *p* ≤ 0.001.

Expression analysis for the BMP type I receptors in HAECs revealed that BMP9 and BMP10 induced the expression of *ALK1* and *ALK2*, with little effect on ALK3 (supplemental Fig. S3, *A–C*). *ALK6* was not expressed. Addition of TNFα slightly reduced the expression of *ALK1*, but not *ALK2*. To determine the BMP type I receptors mediating the BMP9- and BMP10-induced up-regulation in adhesion molecules in response to TNFα, we performed siRNA knockdown of *ALK1* and *ALK2* and assessed surface expression of adhesion molecules. The dependence of each adhesion molecule on ALK1 or ALK2 was different. The increase in cell surface E-selectin expression observed in HAECs pre-treated with BMP9 or BMP10 prior to TNFα was inhibited by *ALK2* knockdown, but not *ALK1* knockdown (Fig. 4*A*), suggesting a marked ALK2 dependence of E-selectin regulation by BMP9 or BMP10 in these experiments. Knockdown of *ALK1* together with *ALK2* did not impact further on E-selectin expression. For VCAM-1 individual siRNA knockdown of *ALK1* and *ALK2* substantially impaired BMP9- and BMP10-induced VCAM-1 expression, and their combined knockdown further inhibited surface VCAM-1 expression (Fig. 4*B*). For ICAM-1, only combined *ALK1* and *ALK2* knockdown resulted in impaired BMP9- and BMP10-induced surface ICAM-1 expression (Fig. 4*C*). The knockdown efficiency of si*ALK1* and si*ALK2* in HAECs confirmed >85% reduction in mRNA levels of the corresponding target gene (supplemental Fig. S3, *D* and *E*). We also confirmed that si*ALK1*, but not si*ALK2*, reduced the *ID1* induction by BMP9 and BMP10 in HAECs (supplemental Fig. S4). These data show that ALK2 is essential for BMP9- and BMP10-induced E-selectin expression, whereas either ALK1 or ALK2 can increase VCAM-1 expression. ICAM-1 requires both ALK1 and ALK2 for up-regulation in TNFα-stimulated HAECs.

**Figure 5.**
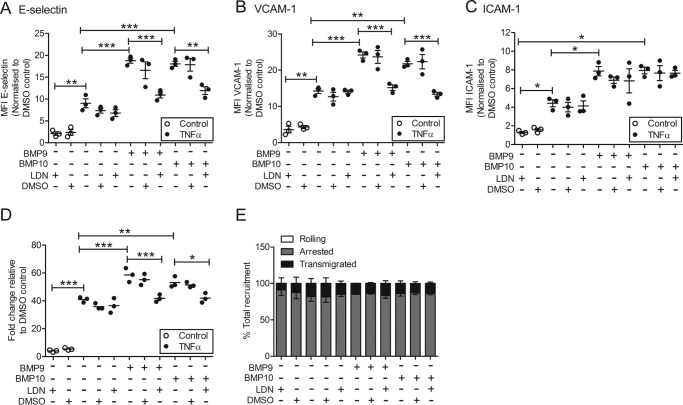
**LDN193189 reduces the BMP9- and BMP10-induced up-regulation of E-selectin, VCAM-1, and ICAM-1 and monocyte recruitment in TNFα-stimulated HAECs.** HAECs were pre-treated with LDN193189 (250 nm resuspended in DMSO), DMSO, then stimulated with BMP9 or BMP10 (5 ng/ml; 16 h) prior to TNFα treatment (0.05 ng/ml for 4 h). Surface expression of E-selectin (FITC-conjugated anti-human E-selectin) (*A*), VCAM-1 (PE-Cy5-conjugated anti-human VCAM-1) (*B*), and ICAM-1 (APC-conjugated anti-human ICAM-1) (*C*) was assessed using flow cytometry. Forward scatter and side scatter gating was applied to the HAEC population. Data are shown as median fluorescence intensity (*MFI*) expressed as fold-change relative to HAECs transfected with siRNA control pool (*siCP*). *D,* HAECs were treated as described above and then monocytes were perfused in a flow adhesion assay. *E,* monocyte behavior (rolling, *clear bar*; adherence, *gray bar*; and transmigration, *black bar*) was expressed as a percentage of total recruitment. Experiments were performed in triplicate and the data are representative of *n* = 3 biological repeats. Error bars represent mean ± S.E. *, *p* ≤ 0.05; **, *p* ≤ 0.01; ***, *p* ≤ 0.001.

To explore further the role of type I receptors in the BMP9/BMP10-induced expression of adhesion molecules in TNFα-stimulated HAECs, we employed LDN193189, a cell permeable small molecule inhibitor of BMP type I receptors. LDN193189 inhibits ALK2 with an IC_50_ of 5 nm, and ALK3 with an IC_50_ of 30 nm, but has no effect on ALK1 in cells (52). LDN193189 also inhibits ACTR-IIA and ACTR-IIB (53). LDN193189 did not affect basal responses. However, pre-treatment of HAECs with LDN193189 decreased the BMP9- or BMP10-induced up-regulation of E-selectin and VCAM-1 (Fig. 5, *A* and *B*). ICAM-1 surface expression levels were only slightly decreased after LDN193189 treatment (Fig. 5*C*). Furthermore, LDN193189 reduced monocyte recruitment induced by BMP9 or BMP10 treatment to the level of TNFα-only stimulation (Fig. 5, *D* and *E*).

### The role of BMP type II receptors in the BMP9- and BMP10-induced expression of adhesion molecules

**Figure 6.**
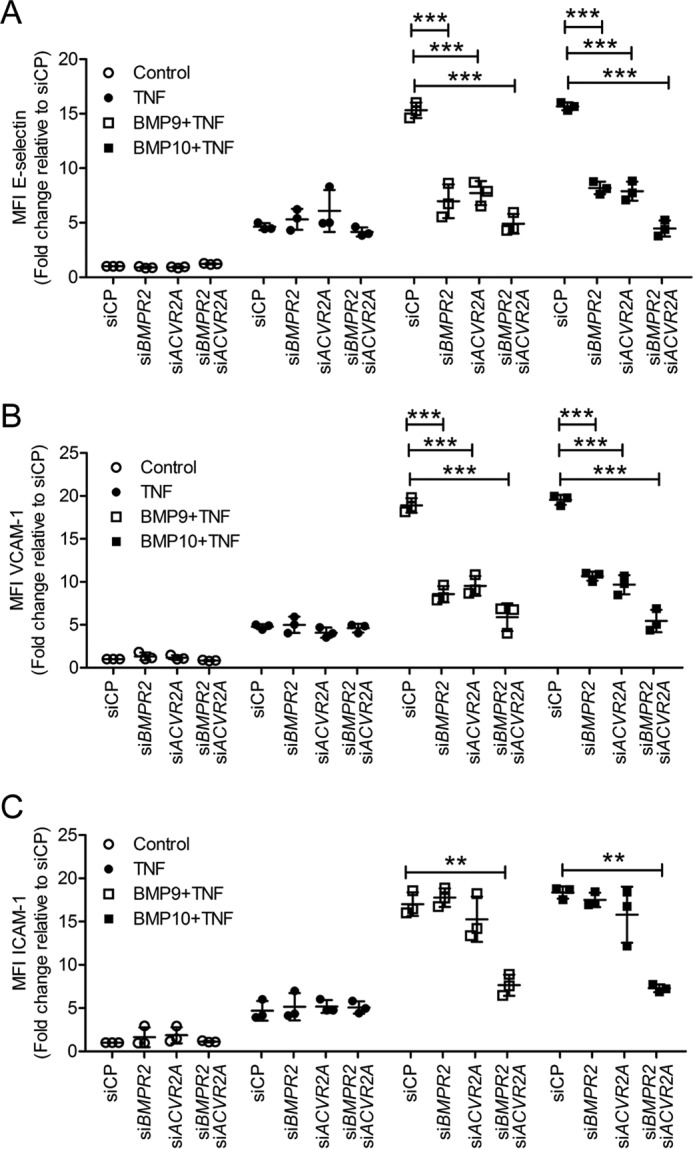
**Knockdown of BMPR2 and ACVR2A inhibits the BMP9- and BMP10-induced up-regulation of E-selectin, VCAM-1, and ICAM-1 in TNFα-stimulated HAECs.** HAECs were siRNA transfected, then treated with BMP9 or BMP10 (5 ng/ml; 16 h) prior to TNFα treatment (0.05 ng/ml; 4 h). Surface expression of E-selectin (FITC-conjugated anti-human E-selectin) (*A*), VCAM-1 (PE-Cy5-conjugated anti-human VCAM-1) (*B*), and ICAM-1 (APC-conjugated anti-human ICAM-1) (*C*) was assessed using flow cytometry. Forward scatter and side scatter gating was applied to the HAEC population. Data are expressed as median fluorescence intensity (*MFI*) expressed as fold-change relative to HAECs transfected with siRNA control pool (*siCP*). Experiments were performed in triplicate and the data are representative of *n* = 3 biological repeats. *Error bars* represent mean ± S.E. *, *p* ≤ 0.05; **, *p* ≤ 0.01; ***, *p* ≤ 0.001.

As *BMPR2* deficiency is associated with pulmonary arterial hypertension (32, 33) and more recently with atherosclerosis (35), we investigated the role of the BMP Type II receptors in mediating the expression of adhesion molecules. Expression analysis revealed that BMP9 and BMP10 induced the expression of *BMPR2*, but not *ACVR2A* or *ACVR2B* (supplemental Fig. S5, *A–C*). TNFα slightly, but nonsignificantly reduced the expression of *BMPR2* (supplemental Fig. S5*A*). Transfection of si*BMPR2* and si*ACVR2A*, both individually and in combination, attenuated the BMP9- and BMP10-induced expression of E-selectin (Fig. 6*A*) and VCAM-1 (Fig. 6*B*) in TNFα-stimulated HAECs. In contrast, individual knockdown of *BMPR2* and *ACVR2A* had no impact on ICAM-1 expression, whereas combined knockdown of these receptors did impair ICAM-1 expression (Fig. 6, *A–C*). The knockdown efficiency for si*BMPR2* and si*ACVR2A* in HAECs were again >85% (supplemental Fig. S5, *D* and *E*).

### Smad1 and Smad5 mediate the BMP9- and BMP10-induced expression of adhesion molecules on TNFα-treated HAECs

**Figure 7.**
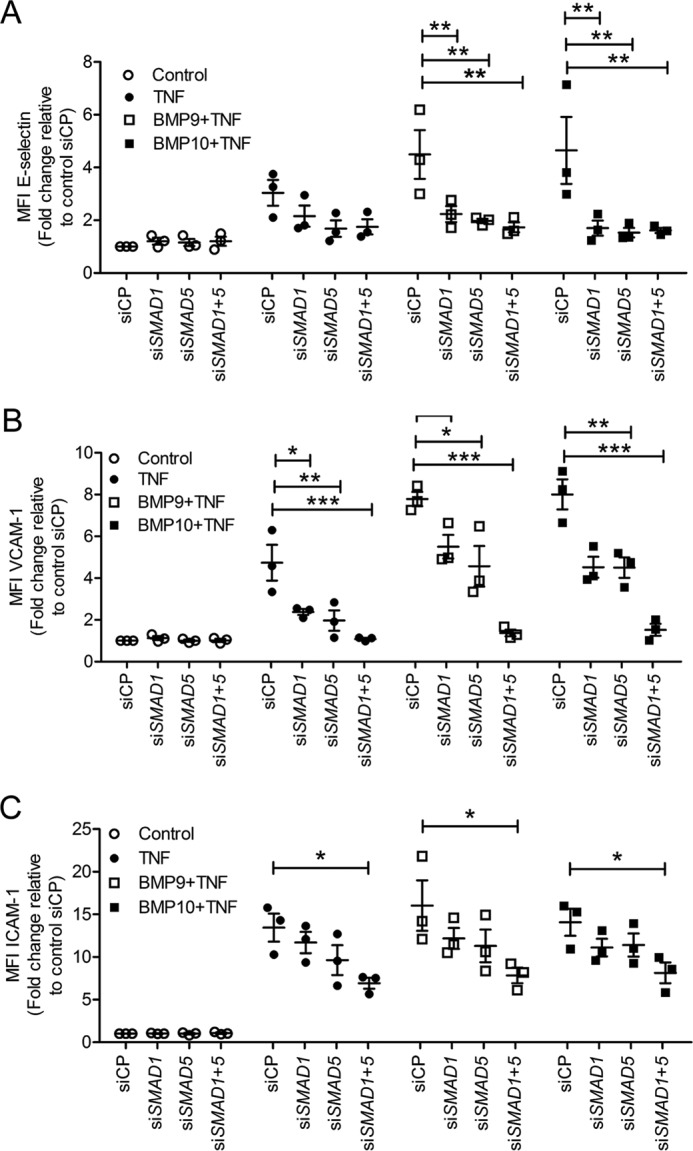
**Smad1 and Smad5 mediate the BMP9- and BMP10-induced up-regulation of E-selectin, VCAM-1, and ICAM-1 in TNFα-stimulated HAECs.** HAECs were siRNA transfected, then treated with BMP9 or BMP10 (5 ng/ml, 16 h) prior to TNFα treatment (0.05 ng/ml, 4 h). Surface expression of E-selectin (FITC-conjugated anti-human E-selectin) (*A*), VCAM-1 (PE-Cy5-conjugated anti-human VCAM-1) (*B*), and ICAM-1 (APC-conjugated anti-human ICAM-1) (*C*) was assessed using flow cytometry. Forward scatter and side scatter gating was applied to the HAEC population. Data are shown as median fluorescence intensity (*MFI*) expressed as fold-change relative to HAECs transfected with siRNA control pool (*siCP*). Experiments were performed in triplicate and the data are representative of *n* = 3 biological repeats. *Error bars* represent mean ± S.E. *, *p* ≤ 0.05; **, *p* ≤ 0.01; ***, *p* ≤ 0.001.

To investigate the involvement of Smad1 and Smad5 (26, 54) in BMP9/BMP10-induced up-regulation of adhesion molecules we employed siRNA knockdown. Unexpectedly, the TNFα-induced expression of VCAM-1, ICAM-1, and E-selectin was inhibited by *SMAD1/5* knockdown, in keeping with the possibility that induction of BMP2 by TNFα was contributing to increased expression of these adhesion molecules (Fig. 7, *A–C*). BMP9- and BMP10-induced expression of E-selectin (Fig. 7*A*) and VCAM-1 (Fig. 7*B*) in TNFα-stimulated HAECs was markedly impaired upon *SMAD1* and *SMAD5* knockdown, both individually and in combination. ICAM-1 expression was only inhibited when *SMAD1* and *SMAD5* were knocked-down in combination (Fig. 7*C*). *SMAD1* and *SMAD5* siRNA knockdown efficiency was confirmed by qPCR and showed an 85% reduction of the target gene (supplemental Fig. S6, *A* and *B*).

Because Smad2 and Smad3 have also been described as mediators of BMP9 signaling (28, 55), we also employed siRNAs targeting *SMAD2* and *SMAD3* (supplemental Fig. S6, *C* and *D*). Knockdown of *SMAD2* and *SMAD3* individually or in combination did not alter the BMP9- or BMP10-induced surface expression of adhesion molecules in HAECs (supplemental Fig. S7). Collectively, these data show that Smad1/5, but not Smad2/3, are essential to the BMP9- and BMP10-induced expression of E-selectin, VCAM-1, and ICAM-1 in TNFα-stimulated HAECs.

### BMP9 and BMP10 increase IκBα protein levels, but do not alter p65/RelA levels or phosphorylation

**Figure 8.**
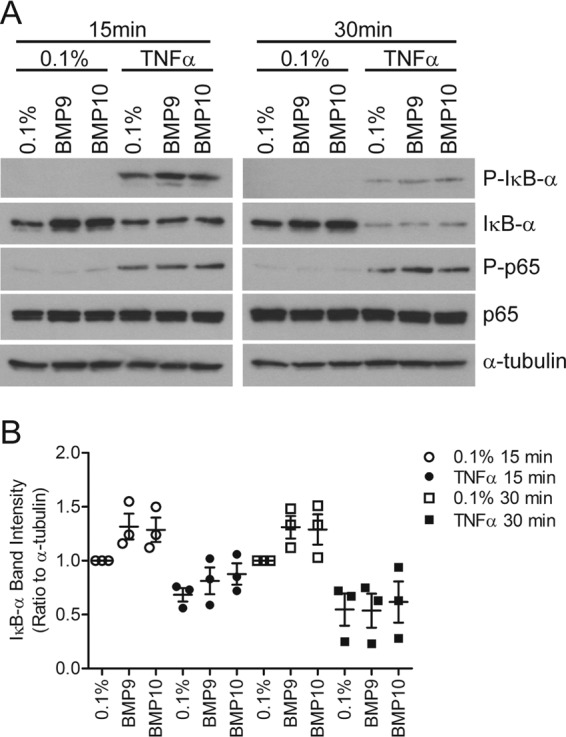
**BMP9 and BMP10 increase IκB-α protein levels in HAECs.** HAECs were treated with BMP9 or BMP10 (5 ng/ml, 16 h) prior to TNFα treatment (0.05 ng/ml) for 15 or 30 min. *A,* protein lysates were immunoblotted for IκB-α, phospho-Ser^32/36^-IκBα (*P-I*κ*B-*α), p65/RelA (*p65*), or phospho-Ser^536^-p65 (*P-p65*). All blots were reprobed for α-tubulin to confirm equal loading. Blots are representative of *n* = 3 separate experiments. *B,* densitometry was determined using ImageJ for the three IκBα blots, each band being expressed as a ratio of IκBα/α-tubulin. These ratios were then normalized to the 0.1% control for the relevant time point.

TNFα is known to mediate the expression of cell surface adhesion receptors via the NF-κB pathway (10). We examined whether BMP9 or BMP10 mediated changes in the levels or phosphorylation of the canonical signaling proteins, IκBα and p65. Both BMP9 and BMP10 increased basal IκBα protein levels (Fig. 8, *A* and *B*), without any impact on IκBα phosphorylation or on levels or serine 536 phosphorylation of p65. These data suggest that BMP9/10 prime endothelial cells for TNFα responsiveness by increasing IκBα levels.

## Discussion

The present study investigated whether BMP9 or BMP10, important circulating regulators of vascular quiescence, play a role in monocyte recruitment to the vascular endothelium. Although BMP9 or BMP10 alone had no effect on monocyte recruitment, in the presence of TNFα both BMPs synergistically and in a concentration-dependent manner increased monocyte recruitment and transmigration. Using siRNA knockdown of type I receptors and a small molecule inhibitor, we show that these effects are predominantly mediated by ALK2 and also involve BMPR-II, ACT-RIIA, and the downstream signaling intermediaries, Smad1/5.

The potentiation of TNFα-mediated monocyte recruitment was observed only at higher concentrations of BMP9 or BMP10, but was also readily induced by BMP6, a ligand with high affinity for ALK2. Knockdown of *ALK1* and *ALK2* in HAECs demonstrated that the BMP9/10-dependent potentiation of the TNFα-stimulated E-selectin expression was entirely ALK2-dependent, whereas VCAM-1 was partially dependent on each receptor and ICAM-1 was only altered when both *ALK1* and *ALK2* were knocked down. Our data suggest a contribution from both ALK1 and ALK2 receptors in mediating the overall response but a dominance of the ALK2 receptor in the potentiation of the BMP9- or BMP10-induced monocyte recruitment to the TNFα-stimulated HAECs. BMP9 and BMP10 both induced the expression of *ALK1* and *ALK2*. The role of these receptors in atherosclerosis is intriguing, as the expression of both *ALK1* and *ALK2* is induced by HDL (56). Moreover, ALK1 mediates endothelial uptake of LDL, but not oxidized LDL in LDLR-deficient mice, suggesting a role for ALK1 in normal endothelial lipid metabolism rather than the pathogenesis of atherosclerosis (57).

To further investigate the role of type I receptors we employed a small molecule inhibitor of ALK2 and ALK3, LDN193189. LDN193189 weakly inhibits ALK4, ALK5, ALK7, ACTR-IIA, and ACTR-IIB (53) at the concentrations used in this study but does not inhibit ALK1 (52). The results confirm that the monocyte recruitment and induction of E-selectin, VCAM-1, and ICAM-1 induced by BMP9 or BMP10 in TNFα-treated HAECs is largely independent of ALK1. Our findings also show that BMP9 and BMP10 synergize with TNFα to induce expression of BMP2, which is a known regulator of endothelial inflammation and plays a role in atherosclerosis. Increased levels of BMP2 and BMP4 have been observed in atherosclerotic plaques (14–17, 58). Once up-regulated, BMP2 and BMP4 induce an inflammatory phenotype in endothelial cells, which results in leukocyte adhesion *in vitro*. BMP4 is increased in response to a high fat diet (a risk factor for atherosclerosis), which then up-regulates BMP2 levels (15). Furthermore, enhanced BMP2 activity has been implicated in triggering and accelerating vascular calcification (14, 15). We and others have shown previously that TNFα increases endothelial expression of *BMP2* but not *BMP4* (12, 59) and that *BMP2* expression in endothelial cells can be activated by inflammatory stimuli in a NF-κB-dependent manner (59, 60). In our current study neither TNFα, BMP9, nor BMP10 alone impacted on the expression of *BMP2*, *BMP4*, or *BMP6* in HAECs. However, HAECs that were treated with BMP9 or BMP10 in the presence of TNFα showed a synergistic increase in *BMP2* expression, providing further evidence that BMP9 and BMP10 are not themselves pro-inflammatory, but instead, might prime the vascular endothelium to mount a more intense response upon stimulation with an inflammatory cytokine such as TNFα.

Chronic TNFα exposure reduces *BMPR2* expression in endothelial cells and can alter BMP signaling (59). Even in this acute study, low concentrations of TNFα reduced basal *BMPR2* expression, whereas cell surface E-selectin, ICAM-1, and VCAM-1 were enhanced. Moreover, both type II receptors, BMPR-II and ACT-RIIA, are essential for BMP9- and BMP10-induced expression of E-selectin and VCAM-1 in TNFα-stimulated HAECs. BMPR-II has been implicated previously in leukocyte-endothelial interactions (38, 39), and *BMPR2* deficiency is associated with several inflammatory vascular pathologies including pulmonary arterial hypertension (32, 33) and atherosclerosis (35). However, there is limited previous information on the role of ACT-RIIA in the regulation of endothelial-expressed selectins or adhesions molecules or the process of leukocyte recruitment (28). These findings provide further insight into the role of endothelial-expressed BMP type II receptors in maintaining endothelial homeostasis.

We questioned whether the mechanism of the enhanced TNFα response in the presence of BMP9/10 might be due to their effect on the NF-κB pathway, the main pathway known to induce endothelial adhesion molecules (10). We identified that BMP9/10 increased IκBα protein levels, but that the rate of IκBα phosphorylation and degradation are not altered. This implies that the cells are primed for the TNF-α response by BMP9/10. Although we did not observe changes in p65/RelA levels or Ser-536 phosphorylation, the NF-κB family members are activated by phosphorylation at several serine residues, so the IκBα priming may be associated with a different family member and/or different phosphorylation sites (61).

In the present study, we have shown that BMP9- and BMP10-induced E-selectin, VCAM-1, and ICAM-1 expression in TNFα-stimulated HAECs is regulated through the canonical BMP mediators, Smad1/5 and not Smad2/3. This correlates with our previous study that reported that Smad1/5 activation was required for BMP9-induced expression of E-selectin and VCAM-1 in LPS-stimulated endothelial cells (37). Smad1/5 has also been reported to mediate the expression of pro-atherogenic genes that promote atherosclerotic plaque stability in monocyte-derived macrophages (62). Moreover, inhibition of BMP signaling using LDN193189 attenuated Smad1/5 activation and reduced endothelial inflammation and calcification in atherosclerosis mouse models (63, 64), thus further supporting our findings that Smad1/5 plays a key role in regulating endothelial homeostasis through the expression of selectins and adhesion molecules.

Although BMP9 has been more extensively characterized than BMP10, there is evidence to suggest that BMP9 and BMP10 can perform overlapping roles. This has been seen *in vitro* whereby BMP9 and BMP10 regulate the expression of a similar set of genes in human microvascular endothelial cells (24). Furthermore, both BMP9 and BMP10 are required for complete closure of the ductus arteriosus (65), and BMP10 can compensate for the absence of BMP9, in BMP9 knock-out mice during retinal vascularization (21). However, Chen and colleagues (66) have shown that BMP9 is not able to substitute for BMP10 during cardiac development in mice, indicating a distinct role for BMP10 in cardiogenesis.

Monocyte transmigration across the endothelium is a normal physiological process but this process can lead to vascular pathologies and promote atherosclerosis if exaggerated. Here we show that treatment alone with either BMP9 or BMP10 (even at concentrations ≥1.5 ng/ml) had no impact on monocyte recruitment in a flow adhesion assay. However, concentrations ≥1.5 ng/ml of BMP9 and BMP10 behave in a near identical manner to synergize with TNFα to up-regulate BMP2 expression and to enhance monocyte adhesion and transmigration in HAECs predominantly through ALK2, BMPR-II/ACT-RIIA, and Smad1/5 signaling. We propose that the beneficial effects of BMP9 or BMP10 as vascular quiescent factors could be subverted in the presence of inflammatory mediators such as TNFα (59), contributing to pathological levels of monocyte recruitment; this in turn might stimulate foam cell development, inflammatory cytokine production, and atherosclerotic plaque development and calcification. Our findings provide further insight into how BMP signaling mediates endothelial homeostasis and the mechanisms by which BMPs impact on cardiovascular disease.

## Experimental procedures

### Reagents, primers, and antibodies

Cell culture reagents were BMP6, BMP9, and BMP10 (R&D Systems) and LDN193189 used at a working concentration of 250 nm (stock resuspended in DMSO at 5 mm, a kind gift from Professor Paul Yu, Department of Medicine, Harvard University) EGM-2 BulletKit (Lonza), fetal bovine serum (FBS) (Sigma), trypsin (Sigma), Histopaque 1077 and 1119 (Sigma), Dulbecco's phosphate-buffered saline (PBS) with Ca^2+^,Mg^2+^ (Sigma), albumin bovine fraction (BSA) V solution 7.5% (Sigma), magnetic-activated cell sorting (MACS) separation system (Miltenyi Biotec), LS columns (Miltenyi Biotec), and CD14 microbeads (Miltenyi Biotec). siRNA transfection reagents were DharmaFECT1^TM^ (Dharmacon), ON-TARGETplus^TM^ siRNA Pools (Dharmacon), namely si*ALK1*, si*ALK2*, si*SMAD1*, si*SMAD2*, si*SMAD3*, si*SMAD5*, si*BMPR2*, si*ACVR2A*, si*ACVR2B,* and non-targeting siRNA Pool (siCP). Flow cytometry reagents were anti-hE-selectin fluorescein-conjugated mouse IgG1(anti-human E-selectin-FITC, R&D Systems), allophycocyanin (APC) mouse anti-human CD54 (anti-human ICAM-1-APC, BD Pharmingen), and PE/Cy5 anti-human CD106 (anti-human VCAM-1-PECy5, BioLegend). Flow cytometry isotype control antibodies: mouse IgG1 isotype control fluorescein (R&D Systems), APC-mouse IgG1 (BD Pharmingen), and PE/Cy5 mouse IgG1 isotype control (BioLegend). Western blotting antibodies were IκBα mouse mAb, phospho-Ser^32/36^-IκBα rabbit Ab, p65/RelA rabbit mAb, or phospho-Ser^536^-p65 rabbit Ab (Cell Signaling Technologies). qPCR reagents were QuantiTect Primer Assays (Qiagen) namely Hs-*ACVRL1_*1_SG (ALK1), Hs_*ACVR1_*1_SG (ALK2), *SMAD2*, *SMAD3*, and *ACVR2A*. Primer sequences were *BMPR2* forward, 5′-CAAATCTGTGAGCCCAACAGTCAA-3′; *BMPR2* reverse, 5′-GAGGAAGAATAATCTGGATAAGGACCAAT-3′; *SMAD1* forward, 5′-TAGAAAGCCCTGTACTTCCTC-3′; *SMAD1* reverse, 5′-GGTTGCTGGAAAGAATCTGG-3′; *SMAD5* forward, 5′-GAGAGTCCAGTCTTACCTCC-3′; *SMAD5* reverse, 5′-GGAAAGAATCTGGAAACGTG-3′; *PBGD* forward, 5′-AGCTATGAAGGATGGGCAAC-3′; *PBGD* reverse, 5′-TTGTATGCTATCTGAGCCGTCTA-3; *B2M* forward, 5′-CTCGCGCTACTCTCTCTTTC-3′; *B2M* reverse, 5′-CATTCTCTGCTGGATGACGTG-3; *HPRT* forward, 5′-GCTATAAATTCTTTGCTGACCTGCTG-3′; *HPRT* reverse, 5′-AATTACTTTATGTCCCCTGTTGACTGG-3. ROX reference dye (Invitrogen) and SYBR Green JumpStart Taq ReadyMix (Sigma) were used.

### Endothelial cell culture

HAECs were purchased from PromoCell and maintained in EGM2-mv (Lonza) with 5% FBS. HAECs were cultured at 37 °C in a 5% CO_2_ humidified atmosphere and used in experiments at passages 4–6. HAECs were treated with BMP6, BMP9, BMP10, or with LDN193189 with the indicated concentrations for 16 h prior stimulation with TNFα (0.05 ng/ml; 4 h).

BOECs were generated from peripheral blood of control volunteers as described previously (46). Full informed written consent was obtained under ethical approval from the Huntington Local Research Ethics Committee.

### Monocyte isolation

Blood samples were derived from healthy volunteers after giving informed consent, according to the protocol approved by the Cambridge Research Ethics Committee (06/Q018/218). Two-step density gradients of Histopaque 1119 and 1077 (Sigma) were used to isolate peripheral blood mononuclear cells. CD14^+^ monocytes were isolated from peripheral blood mononuclear cells through positive selection using magnetic-activated cell sorting as per the manufacturer's instructions. CD14^+^ monocytes were resuspended at a cell density of 1 × 10^6^ cells/ml in 0.15% BSA in PBS (with Ca^2+^ and Mg^2+^).

### siRNA transfection

HAECs were transfected with siRNA at 10 nm final concentration, using DharmaFECT1^TM^ transfection reagent, following the manufacturer's instructions, 48 h prior to their use in cell culture experiments.

### Monocyte-endothelial interactions under flow

An *in vitro* flow adhesion assay was used to assess endothelial–monocyte interactions as previously described (67). The microslide (μ-Slide VI^0.4^; Ibidi), containing the HAEC monolayer was connected to cell and wash reservoirs by silicon tubing and a valve enabled switching between the two reservoirs with continuous flow. The flow rate of 1 × 10^6^ monocytes/ml for 4 min, equivalent to a wall shear stress of 0.1 Pascals, was controlled using a glass syringe attached to a withdrawal pump. Monocyte–endothelial interactions were visualized using time lapse imaging at 6 min post the initial monocyte bolus using a phase-contrast microscope, placed within a Perspex environmental chamber at 37 °C. Quantification of monocyte behavior including rolling, arrest, and transmigration was performed offline using ImagePro software.

### Flow cytometric analysis of surface proteins

Flow cytometric analysis of endothelial cell surface adhesion proteins was performed as previously described (34) using anti-human E-selectin-FITC, anti-human VCAM-1-PE-Cy5, and anti-human ICAM-APC with corresponding conjugated isotype controls. Analysis was performed using a BD FACSCanto^TM^ II (BD Biosciences) and quantification was performed using FlowJo software.

### qPCR

An RNAeasy Mini kit (Qiagen) was used to extract the total RNA extracted from HAECs. mRNA expression of the genes of interest was assessed using SYBR Green Jumpstart Taq ReadyMix, ROX reference dye, and primers (Quantitect Primer Assays or in-house designed primers) in a 384-well QuantStudio 6 Flex (Applied Biosystems, Life Technologies). The ΔΔ*C_t_* method was used for quantification.

### Western blotting

HAECs were seeded in 6-cm dishes and grown to confluence. Cells were then incubated in EBM2 (Lonza) with 0.1% FBS (0.1% FBS) for 2 h and then treated with BMP9, BMP10, or 0.1% FBS for 16 h. Cells were then treated with TNFα (0.05 ng/ml) or 0.1% FBS for 15 or 30 min. Cells were snap-frozen and lysed in 250 mm Tris-HCl, pH 6.8, 4% SDS, 20% (v/v) glycerol containing an EDTA-free protease inhibitor mixture (Roche Applied Science, West Sussex, UK). Lysates were immunoblotted for the relevant proteins.

### Statistical analysis

Comparisons between two groups were made using an unpaired Student's *t* test. Comparisons between three or more groups were performed using one-way analysis of variance with Tukey's multiple comparisons. A probability (*p* value) smaller than 0.05 was considered statistically significant. Normality of data distribution was assessed using a D'Agostino and Pearson omnibus normality test. Data are presented as the mean ± S.E.

## Author contributions

C. G. M designed and performed the research, analyzed the results, and wrote the paper. S. L. A. designed the research and wrote the paper. G. B. N. wrote the paper. Z. M. wrote the paper. E. R. C. wrote the paper. P. D. U. designed the research and wrote the paper. N. W. M. designed the research, analyzed the data, and wrote the paper.

## Supplementary Material

Supplemental Data

## Footnotes

4 The abbreviations used are:

ICAM-1: intercellular adhesion molecule 1
VCAM-1: vascular cell adhesion molecule 1
BMP: bone morphogenetic protein
Alk: activin-like kinase
HAEC: human aortic endothelial cell
BOEC: blood outgrowth endothelial cell
qPCR: quantitative PCR
APC: allophycocyanin.
