# Supplementary material for: Bone morphogenetic protein 9 (BMP9) and BMP10 enhance tumor necrosis factor-α-induced monocyte recruitment to the vascular endothelium mainly via activin receptor-like kinase 2
Source: J Biol Chem. 2017 Jun 23;292(33):13714–26. doi: 10.1074/jbc.M117.778506 (PMC5566526; doi:10.1074/jbc.M117.778506)
Supplement: Supplemental Data [file supp_292_33_13714__index.html]

Bone morphogenetic protein (BMP) 9 and BMP10 enhance tumor necrosis factor-α-induced monocyte recruitment to the vascular endothelium mainly via activin receptor-like kinase 2. — Bone morphogenetic protein 9 (BMP9) and BMP10 enhance tumor necrosis factor-α-induced monocyte recruitment to the vascular endothelium mainly via activin receptor-like kinase 2 — BMP9/BMP10 increases monocyte recruitment to endothelium — Supplemental Data 

# Bone morphogenetic protein 9 (BMP9) and BMP10 enhance tumor necrosis factor-α-induced monocyte recruitment to the vascular endothelium mainly via activin receptor-like kinase 2

## Supplemental Data

- Supplemental data (.docx, 2.4 MB) - File containing supplement figures S1-7
