# Supplementary material for: Bone morphogenetic protein 9 (BMP9) and BMP10 enhance tumor necrosis factor-α-induced monocyte recruitment to the vascular endothelium mainly via activin receptor-like kinase 2
Source: J Biol Chem. 2017 Jun 23;292(33):13714–26. doi: 10.1074/jbc.M117.778506 (PMC5566526; doi:10.1074/jbc.M117.778506)
Supplement: Supplemental Data [file 10.1074_M117.778506_jbc.M117.778506-1.docx]

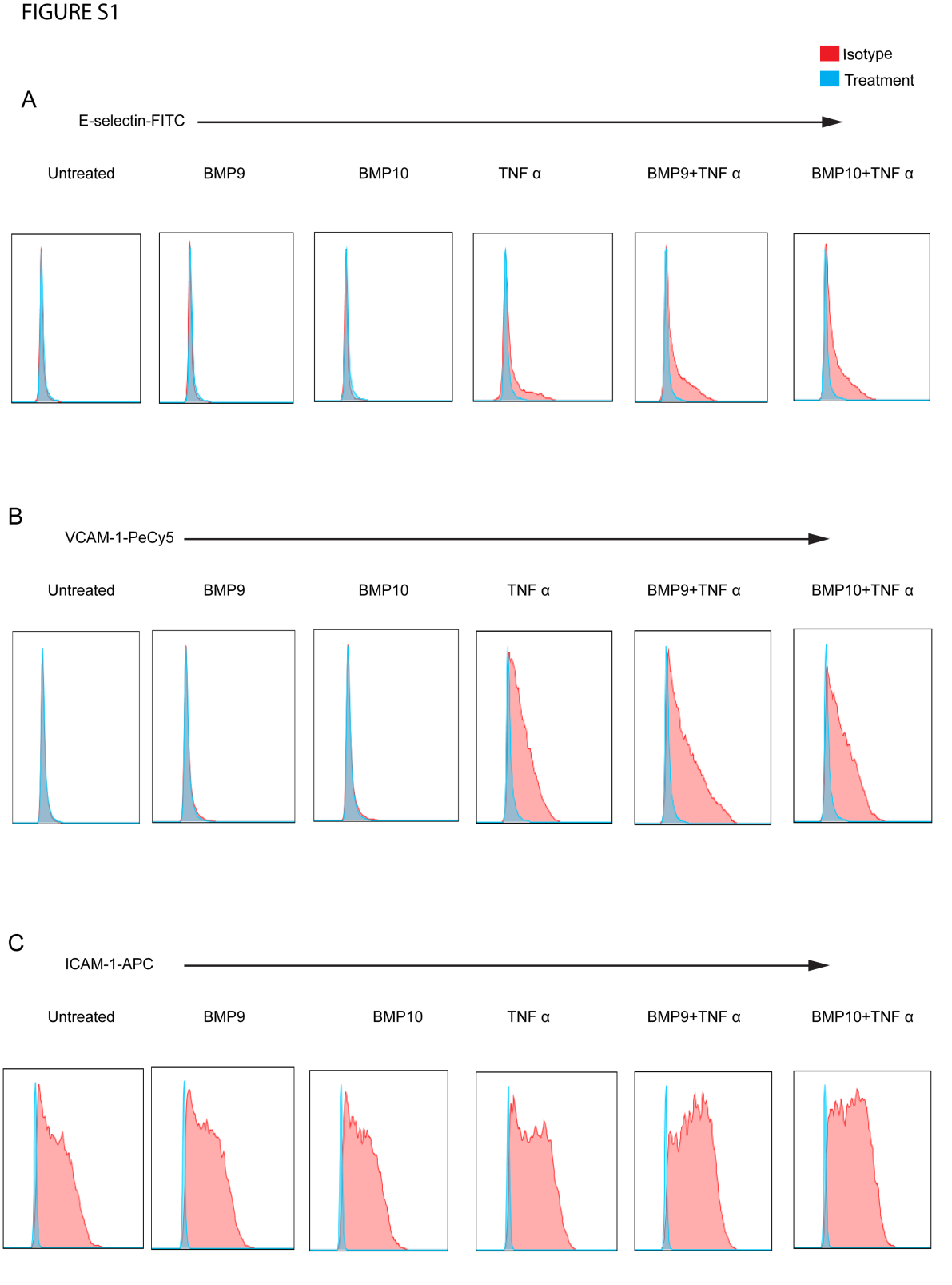


FIGURE S1. **Representative flow cytometric histograms showing the cell surface expression of E-selectin, VCAM-1 and ICAM-1 in human aortic endothelial cells (HAECs).** HAECs were treated with BMP9 or BMP10 (5ng/ml, 16 h) followed by TNFα stimulation (0.05ng/ml, 4 h). Flow cytometry was performed to assess surface expression of **(A)** E-selectin (FITC-conjugated anti-human E-selectin), **(B)** VCAM-1 (PE-Cy5-conjugated anti-human VCAM-1) and **(C)** ICAM-1 (APC-conjugated anti-human ICAM-1) in HAECs. Forward scatter and side scatter gating was applied to the HAEC population. Histograms are representative of n=3 experiments.


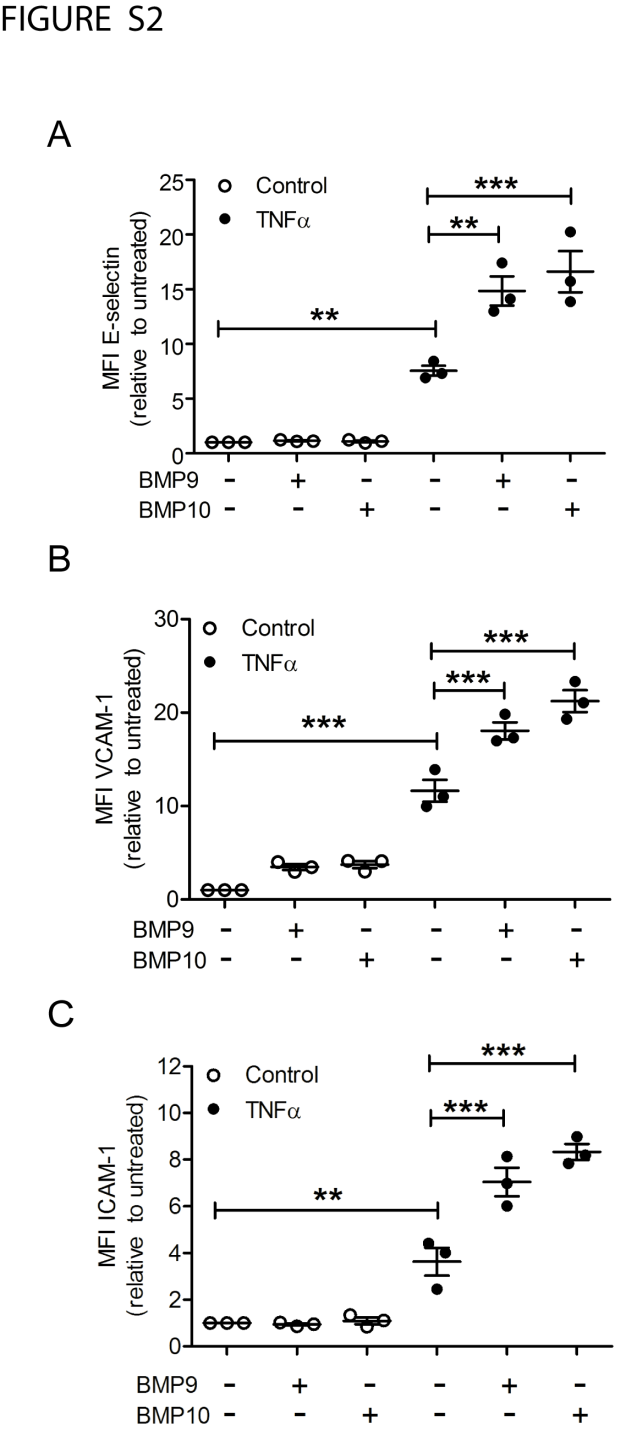


FIGURE S2. **BMP9 and BMP10 increase the surface expression of E-selectin, VCAM-1 and ICAM-1 in TNFα-stimulated human blood outgrowth endothelial cells (BOECs).** BAECS were treated with BMP9 or BMP10 (5ng/ml, 16 h) prior to TNFα treatment (0.05ng/ml, 4 hours). Surface expression of **(A)** E-selectin (FITC-conjugated anti-human E-selectin), **(B)** VCAM-1 (PE-Cy5-conjugated anti-human VCAM-1) and **(C)** ICAM-1 (APC-conjugated anti-human ICAM-1) was assessed using flow cytometry. Data are shown as median fluorescence intensity (MFI) expressed as fold change relative to untreated BOECs. Experiments were performed in triplicate and the data are representative of n=3 biological repeats. Error bars represent ± S.E.M. **P ≤ 0.01, ***P ≤ 0.001.


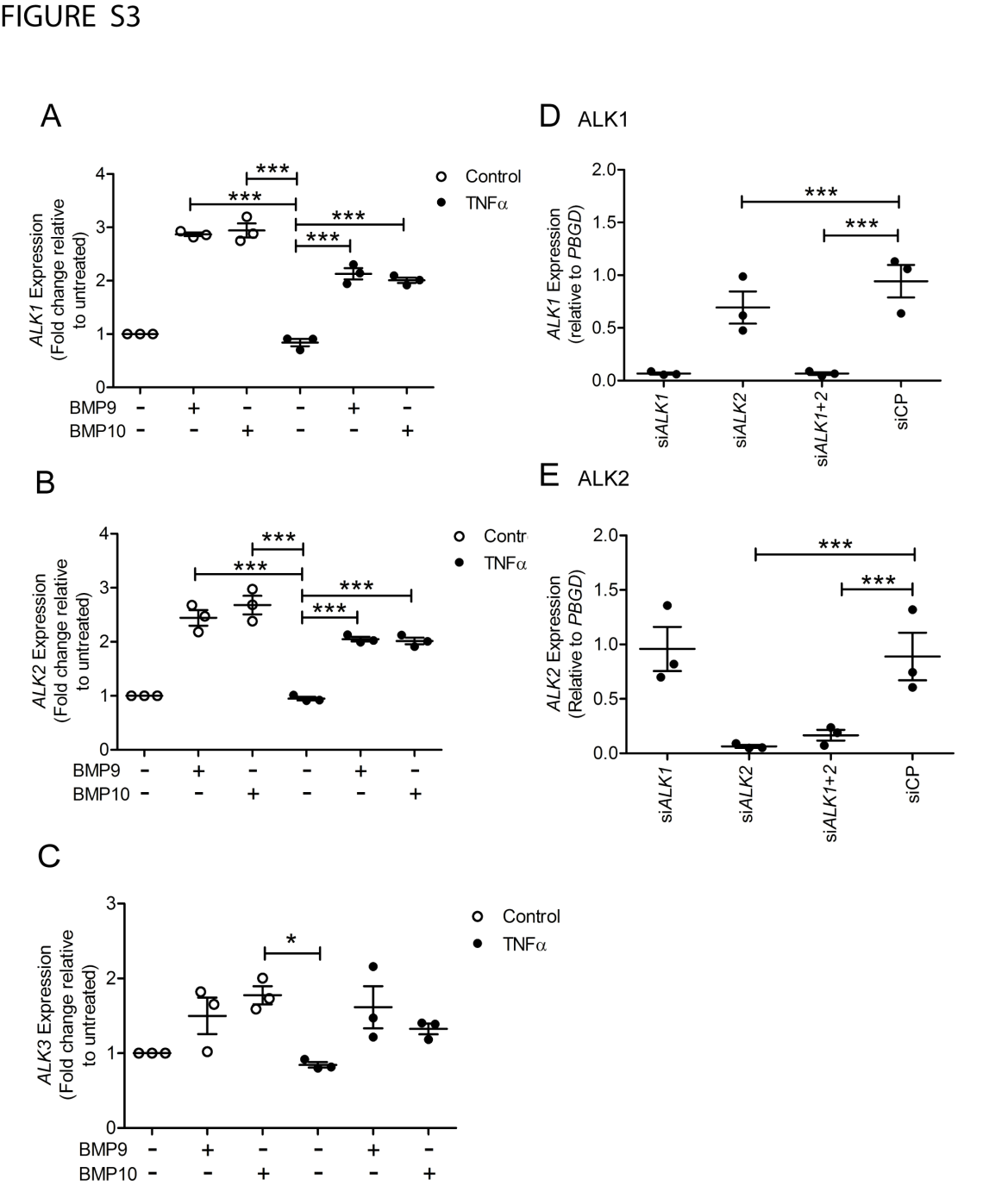


FIGURE S3. **ALK Gene Expression in HAECs. (A-C)** HAECS were treated with BMP9 or BMP10 (5ng/ml, 16 h) prior to TNFα treatment (0.05ng/ml, 4 hours). Expression of **(A)** *ALK1*, **(B)** *ALK2* and **(C)** *ALK3* mRNA levels assessed using qRT-PCR mRNA and normalised to the untreated control. **(D-E)** Assessment of the reduction of **(D)** *ALK1* and **(E)** *ALK2* mRNA in HAECs transfected with siRNAs for these receptors. Levels of mRNA were determined relative to HAECs transfected with the control pool (siCP). Error bars represent ± S.E.M. *P ≤ 0.05, ***P ≤ 0.001.


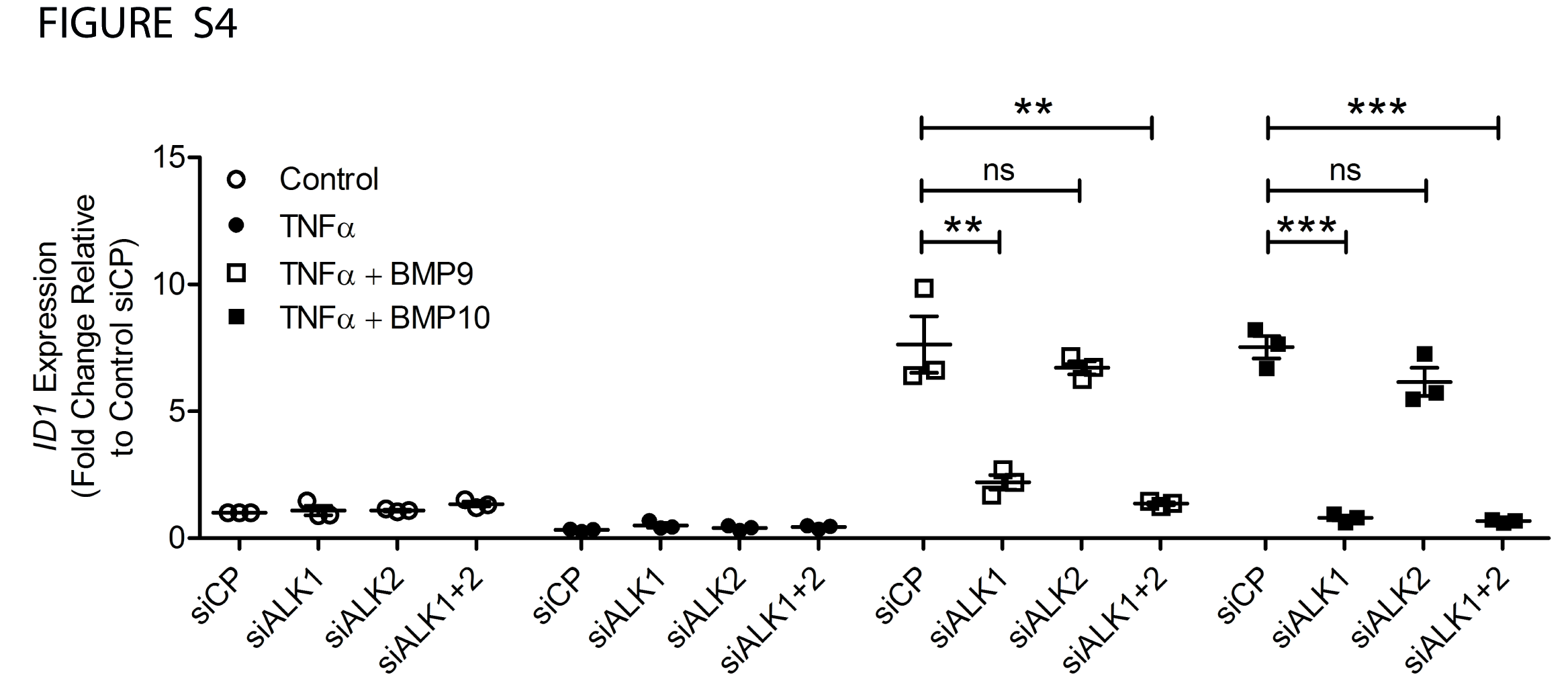


FIGURE S4. **BMP9 and BMP10 Mediate *ID1* Gene Expression Via ALK1 in HAECs.** HAECS were transfected with si*ALK1*, si*ALK2* or both in combination. Cells were then treated with BMP9 or BMP10 (5ng/ml, 16 h) prior to TNFα treatment (0.05ng/ml, 4 hours). Expression of *ID1* mRNA levels were assessed using qRT-PCR mRNA. Experiments were performed in triplicate and the data are representative of n=3 biological repeats. Error bars represent ± S.E.M. **P ≤ 0.01, ***P ≤ 0.001.


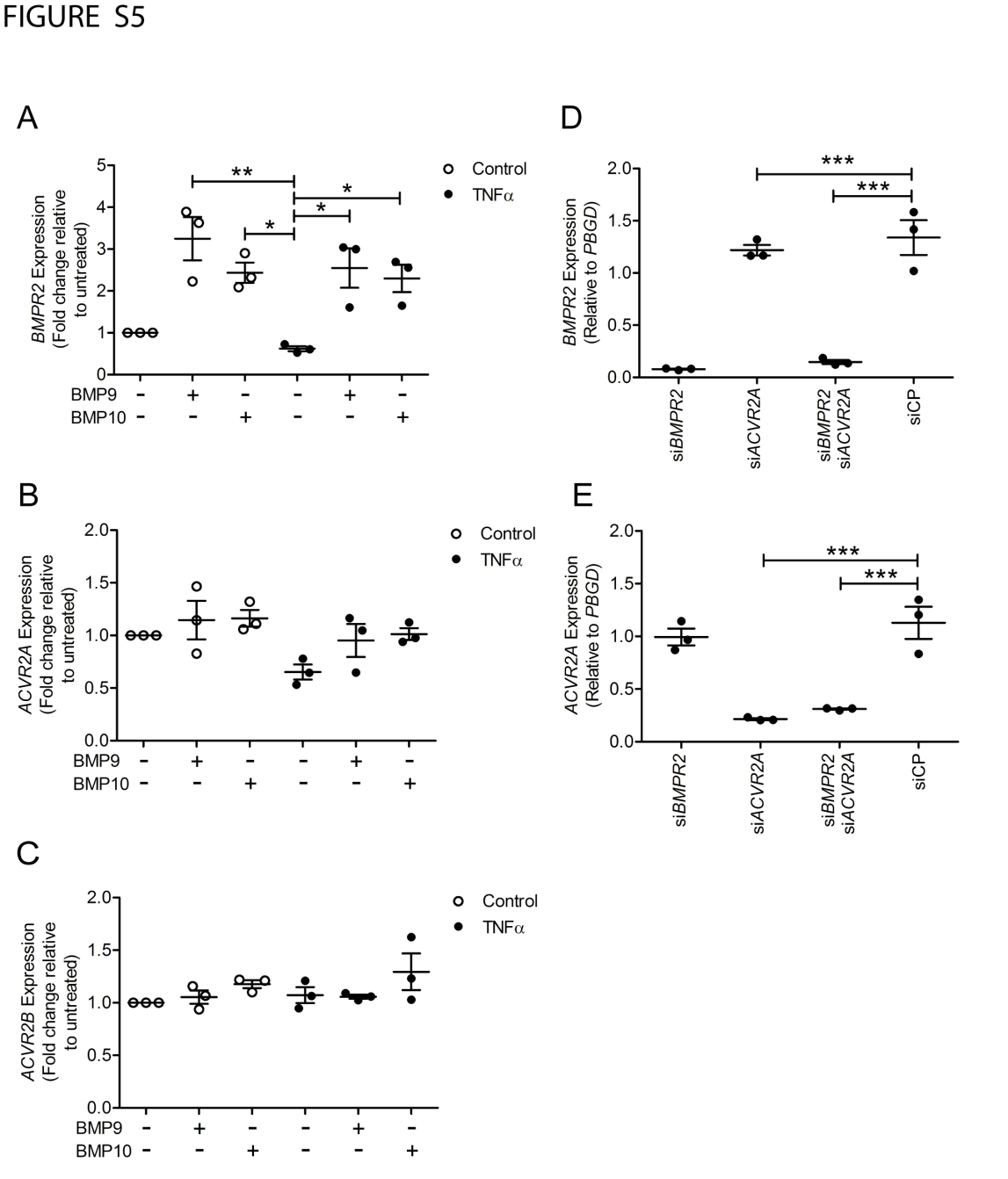


FIGURE S5. **BMP Type-II Receptor Expression in HAECs. (A-C)** HAECS were treated with BMP9 or BMP10 (5ng/ml, 16 h) prior to TNFα treatment (0.05ng/ml, 4 hours). Expression of **(A)** BMPR2, **(B)** *ACVR2A* and **(C)** *ACVR2B* mRNA levels assessed using qRT-PCR mRNA and normalised to the untreated control. **(D-E)** Assessment of the reduction of **(D)** *BMPR2* and **(E)** *ACVR2A* mRNA in HAECs transfected with siRNAs for these receptors. Levels of mRNA were determined relative to HAECs transfected with the control pool (siCP). Error bars represent ± S.E.M. *P ≤ 0.05, **P ≤ 0.01, ***P ≤ 0.001.


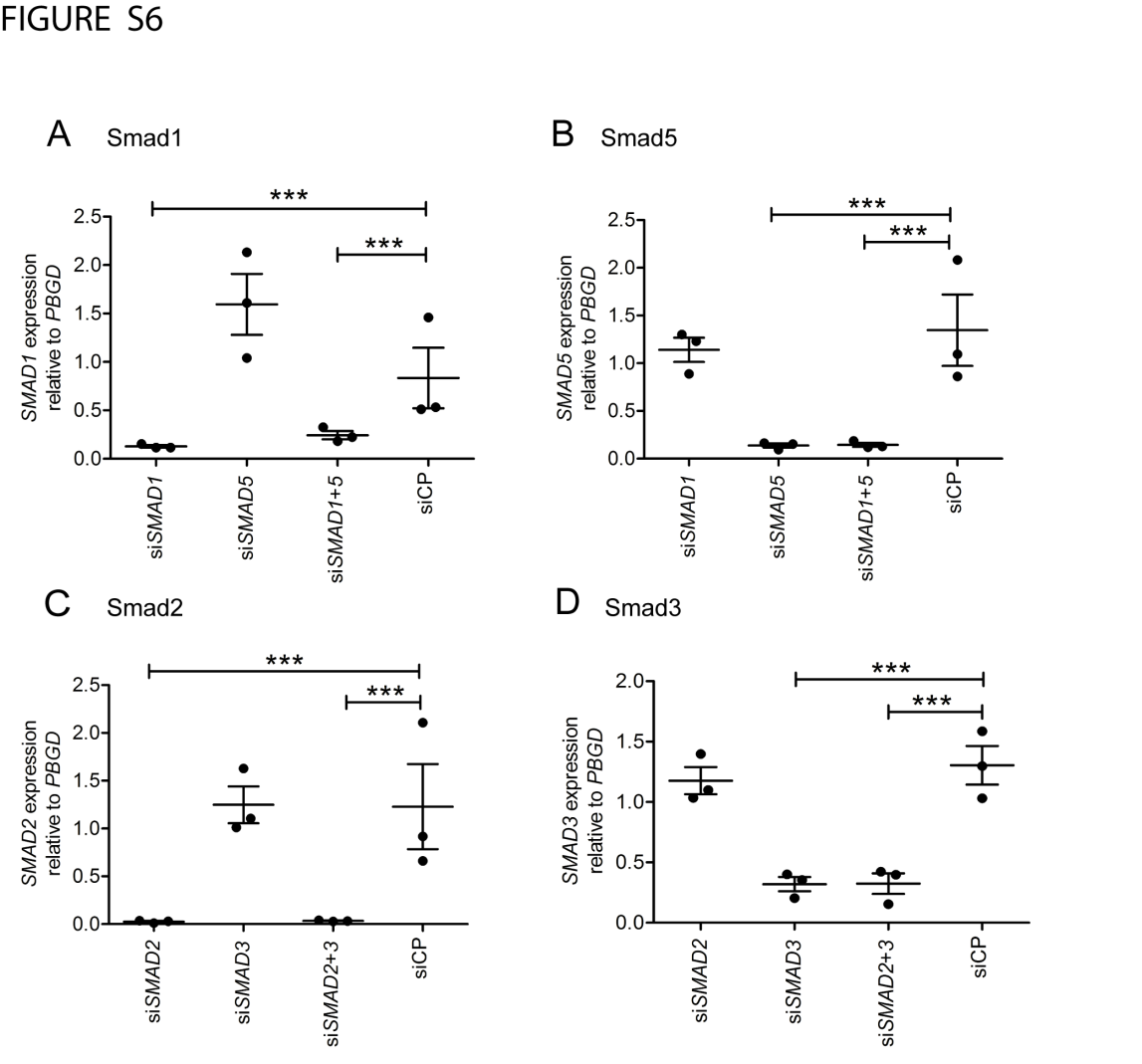


FIGURE S6. **Assessment of SMAD** **Knockdown Efficiency in human aortic endothelial cells (HAECs). (A)** *SMAD1*, **(B)** *SMAD5*, **(C)** *SMAD2* and **(D)** *SMAD3* mRNA levels were quantified as fold expression compared to *PBGD* housekeeping gene and relative to HAECs transfected with the control pool (siCP). Error bars represent ± S.E.M. ***P ≤ 0.001.


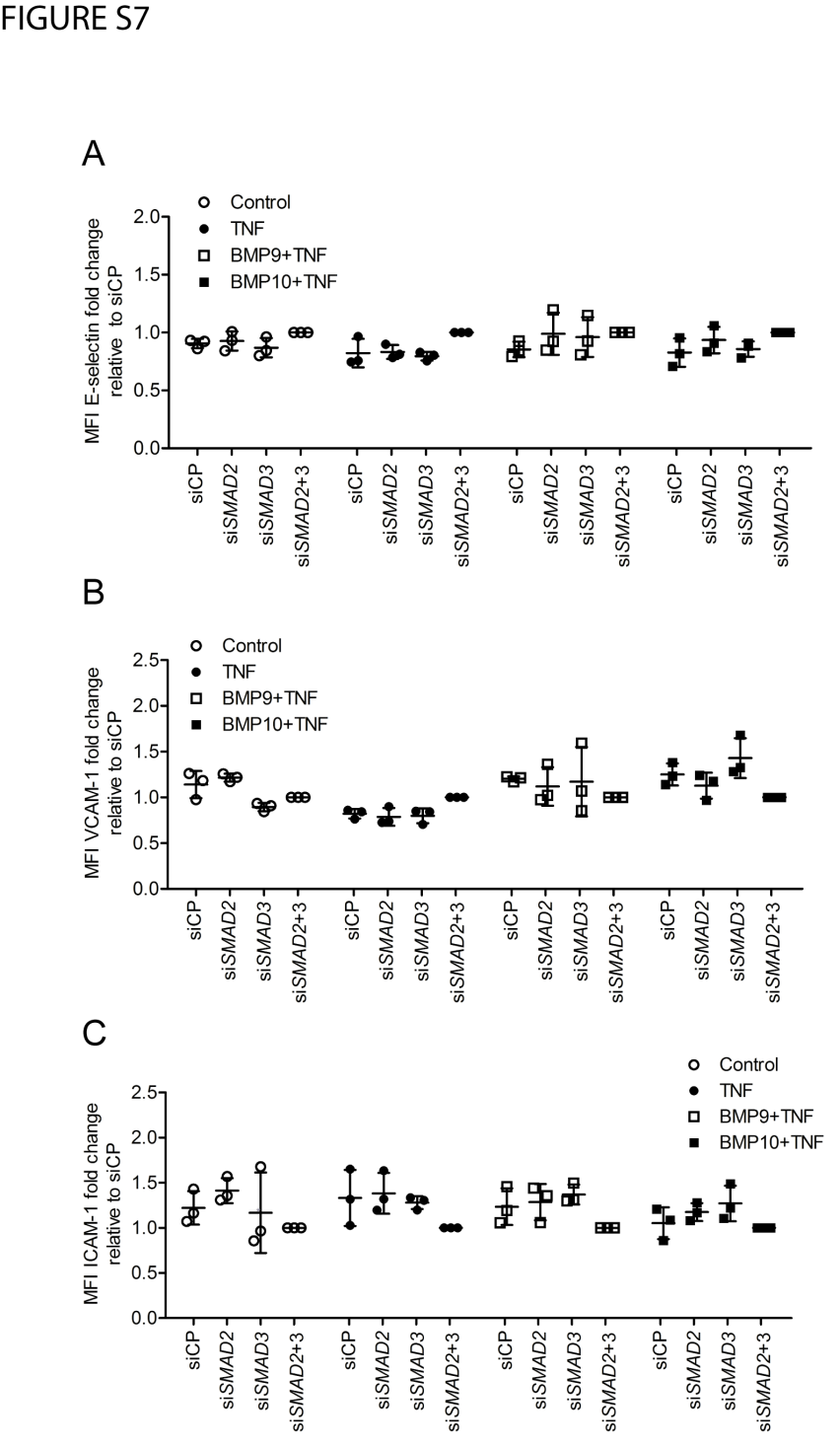


FIGURE S7**. Knockdown of *SMAD2* and *SMAD3* does not affect BMP9- or BMP10-induced upregulation of E-selectin, VCAM-1 or ICAM-1 in TNFα-stimulated human aortic endothelial cells (HAECs).** HAECs were siRNA transfected with si*SMAD2*, si*SMAD3* or both in combination, then treated with BMP9 or BMP10 (5ng/ml for 16 h) prior to TNFα treatment (0.05ng/ml, 4 h). Surface expression of **(A)** E-selectin (FITC-conjugated anti-human E-selectin), **(B)** VCAM-1 (PE-Cy5-conjugated anti-human VCAM-1) and **(C)** ICAM-1 (APC-conjugated anti-human ICAM-1) in HAECs was assessed using flow cytometry. Data are shown as median fluorescence intensity (MFI) expressed as fold change relative to HAECs transfected with siRNA control pool (siCP). Experiments were performed in triplicate and the data are representative of n=3 biological repeats. Error bars represent ± S.E.M.
